# Removal of the Kidneys for Nephro-Lithiasis

**Published:** 1882-03

**Authors:** 


### REMOVAL OF THE KIDNEYS FOR NEPHRO-LITHIASIS.

At the Charing-Cross Hospital is a lad aged fifteen, from whom Mr. Bardwell removed a kidney on May 5th, and who is now convalescent. The boy had been under observation for about a year with pyelitis and retro-peritoneal abscess. An incision was made about ten months ago with the effect of mitigating the symptoms. The wound had healed, leaving only a sinus. In April, by sounding through this passage, Mr. Bardwell detected a stone. Yet, although the lad was becoming very anæmic with irregular hectic temperature, no consent for operation could be obtained until the above date, when lumbar nephrectomy was performed. Two peculiarities rendered the removal unusually difficult, viz.: the dense, thick cicatrical tissue and the proximity of the rib to the hilum. Mr. Bardwell cut through the tissues, and came upon the kidney with the stone impacted. An endeavor to extract this latter caused copious bleeding, hence the operator rapidly enucleated the gland and passed a ligature around the pedicle *en masse*. Since want of room prevented removing the kidney entire, it was divided and extracted in two parts. The operation was thus completed very quickly, and with scarcely any loss of blood. Since then the boy has been going on uninterruptedly well, his temperature becoming normal and regular, the wound being now nearly healed. This is, we believe, the second successful case of the removal of the kidney for stone.—*London Lancet.*

